# Higenamine Combined with [6]-Gingerol Suppresses Doxorubicin-Triggered Oxidative Stress and Apoptosis in Cardiomyocytes via Upregulation of PI3K/Akt Pathway

**DOI:** 10.1155/2013/970490

**Published:** 2013-06-05

**Authors:** Yan-Ling Chen, Xiao-Dong Zhuang, Zhi-Wei Xu, Li-He Lu, Hua-Lei Guo, Wei-Kang Wu, Xin-Xue Liao

**Affiliations:** ^1^Department of Pathophysiology, Zhongshan School of Medicine, Sun Yat-Sen University, 74 Zhongshan Road, Guangzhou 510080, China; ^2^Institute of Integrated Traditional Chinese and Western Medicine, Sun Yat-Sen University, Guangzhou 510080, China; ^3^Department of Cardiology, the First Affiliated Hospital of Sun Yat-Sen University, Guangzhou 510080, China

## Abstract

Sini decoction is a well-known formula of traditional Chinese medicine, which has been used to treat cardiovascular disease for many years. Previously, we demonstrated that Sini decoction prevented doxorubicin-induced heart failure in vivo. However, its active components are still unclear. Thus, we investigated the active components of Sini decoction and their cardioprotective mechanisms in the in vitro neonatal rat cardiomyocytes and H9c2 cell line models of doxorubicin-induced cytotoxicity. Our results demonstrated that treatment with higenamine or [6]-gingerol increased viability of doxorubicine-injured cardiomyocytes. Moreover, combined use of higenamine and [6]-gingerol exerted more profound protective effects than either drug as a single agent, with effects similar to those of dexrazoxane, a clinically approved cardiac protective agent. In addition, we found that treatment with doxorubicin reduced SOD activity, increased ROS generation, enhanced MDA formation, induced release of LDH, and triggered the intrinsic mitochondria-dependent apoptotic pathway in cardiomyocytes, which was inhibited by cotreatment of higenamine and [6]-gingerol. Most importantly, the cytoprotection of higenamine plus [6]-gingerol could be abrogated by LY294002, a PI3K inhibitor. In conclusion, combination of higenamine and [6]-gingerol exerts cardioprotective effect against doxorubicin-induced cardiotoxicity through activating the PI3K/Akt signaling pathway. Higenamine and [6]-gingerol may be the active components of Sini decoction.

## 1. Introduction

Doxorubicin (DOX) is one of the most effective chemotherapeutic agent for the treatment of a wide variety of cancers, including lymphoma, leukemia, and solid tumors [1]. However, some restrictions have been imposed on its clinical use due to its acute and chronic cardiotoxicity. DOX-induced cardiotoxicity is a complex multifactorial process, the mechanisms of which are not completely understood. Most evidence indicates that DOX induces cardiotoxicity through redox cycling and ROS generation [2, 3]. Recently, however, Zhang et al. [4] reported that Top2*β* was required to initiate the entire cardiotoxicity cascade. Regardless of the pathogenesis, exposure of cardiomyocytes to DOX induces ROS generation and causes mitochondrial structural and functional alterations. The increase in oxidative stress and depletion of endogenous antioxidants will trigger the intrinsic mitochondria-dependent apoptotic pathway in cardiomyocytes. Numerous signal molecules, such as cytochrome c, superoxide dismutase, Bcl-2, and Bax, have been indicated in the reactive oxygen species-induced apoptotic pathways of cardiomyocytes [5]. 

**Figure 1:**
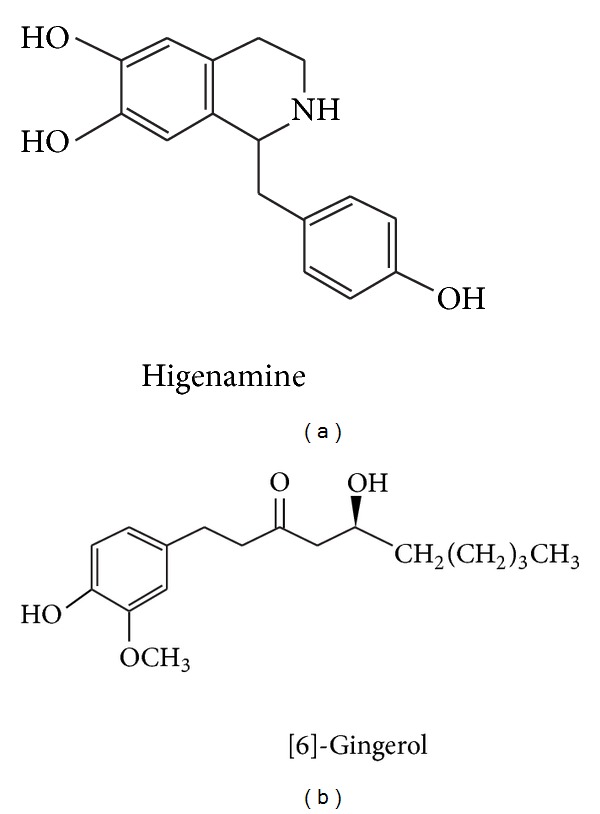
Chemical structures of higenamine (a) and [6]-gingerol (b).

Sini decoction is a well-known formula of traditional Chinese medicine, which has been officially recorded in Chinese Pharmacopoeia 2010 Edition. It is composed of three medicinal herbs: *Aconiti Lateralis Radix Praeparata*, *Rhizoma Zingiberis*, and *Glycyrrhiza uralensis*. Various studies have showed that Sini decoction was an efficient agent against cardiovascular disease [6–8]. Previously, we have proven the protective role of Sini decoction in DOX-induced heart failure [9, 10]. However, little is known about the active components of Sini decoction in DOX-induced cardiac damage. In recent years, more and more compounds have been isolated and identified from Sini decoction. Higenamine (HG), (1-[(4-hydroxyphenyl) methyl]-1,2,3,4-tetrahydroisoquinoline-6,7-diol) (structure shown in Figure 1(a)), an active ingredient of Aconiti Lateralis Radix Praeparata, has been traditionally used as a heart stimulant and anti-inflammatory agent in oriental countries [11]. Studies have showed that HG has protective roles in many cardiovascular diseases via reducing platelet adhesion [12], inhibiting action of iNOS expression [13], and upregulating the expression of HO-1 [14]. In addition, HG exerts positive chronotropic and inotropic effects [15] and possesses antiapoptotic functions mediated by the Akt prosurvival axis in brain cells and C6 cells [11]. The above-mentioned effects suggest that HG would be beneficial for congestive heart failure. Gingerols are considered to be the major constituents of ginger (Rhizoma Zingiberis) which has been used as a popular spice and flavoring agent for a long time all over the world [16]. Among all the gingerols, [6]-gingerol (1-[4′-hydroxy-3′-methoxyphenyl]-5-hydroxy-3-decanone) (structures shown in Figure 1(b)) appears to be responsible for most of the pharmaceutical actions of ginger. [6]-Gingerol has been found to possess diverse interesting pharmacological and physiological effects, such as antioxidant [17], anti-inflammatory [18], and antiplatelet aggregation [19]. In addition, research shows that glycyrrhizin (GC), one of the main active ingredients of *Glycyrrhiza uralensis*, protects rat heart against ischemia-reperfusion injury through blockade of HMGB1-dependent phospho-JNK/Bax pathway [20]. Based on the rationale, we hypothesize that these compounds may be the active components of Sini decoction.

In the present study, we investigated the active components of Sini decoction and the signaling mechanisms underlying the protective role of these components against DOX-induced cardiomyocytes apoptosis and oxidative stress using two in vitro cell models. In addition, we studied the signaling mechanism of the PI3K/Akt pathways, as these pathways play important roles in mediating survival signaling in cardiomyocytes. In this study, we demonstrated that HG/[6]-GR (HG plus [6]-GR) combination exerts cardioprotective effect against doxorubicin-induced cardiotoxicity through the activation of the PI3K/Akt signaling pathway. These findings indicated that HG and [6]-GR may be the active components of Sini decoction, thus providing a promising new strategy for the treatment of DOX-induced cardiotoxicity.

## 2. Materials and Methods

### 2.1. Materials

Doxorubicin was obtained from Shenzhen Wanle Pharmaceutical Co., Ltd. (China). [6]-Gingerol was a reference compound (purity >98%) purchased from Tauto Biotech Co., Ltd (Shanghai, China). Glycyrrhizin (purity >98%) was purchased from Guangzhou Institute for Drug Control. Higenamine (purity >98%) was generously provided by Zhuhai Rundu Mintong Pharmaceutical Co., Ltd. (China). M199, DMEM, trypsin, penicillin, and streptomycin were purchased from Gibco (Invitrogen, Carlsbad, CA, USA). Fetal bovine serum (FBS) and donor equine serum were obtained from Hyclone (Logan, UT, USA). Methylthiazolyldiphenyl-tetrazolium bromide (MTT) was purchased from Sigma (St Louis, MO, USA). 2′, 7′-Dichloro dihydrofluorescein diacetate (DCFH-DA), 5, 5′, 6,  6′-tetrachloro-1, 1′, 3, 3′-tetra ethyl benzimidazole carbocyanine iodide (JC-1) were obtained from Beyotime Institute of Biotechnology (Nantong, China). LY294002, a specific inhibitor of phosphatidylinositol 3-kinase, was purchased from LC Laboratories (Woburn, MA, USA). Antibodies against procaspase-3, cleaved caspase-3, PI3K, total and phospho-Akt, Bax, Bcl-2, and cytochrome c were obtained from Cell Signaling Technology (Boston, MA, USA). *β*-actin antibody was purchased from Sigma (St Louis, MO, USA).

### 2.2. Experiments in Neonatal Rat Cardiomyocytes

#### 2.2.1. Neonatal Rat Cardiomyocytes (NRCs) Culture

**Figure 2:**
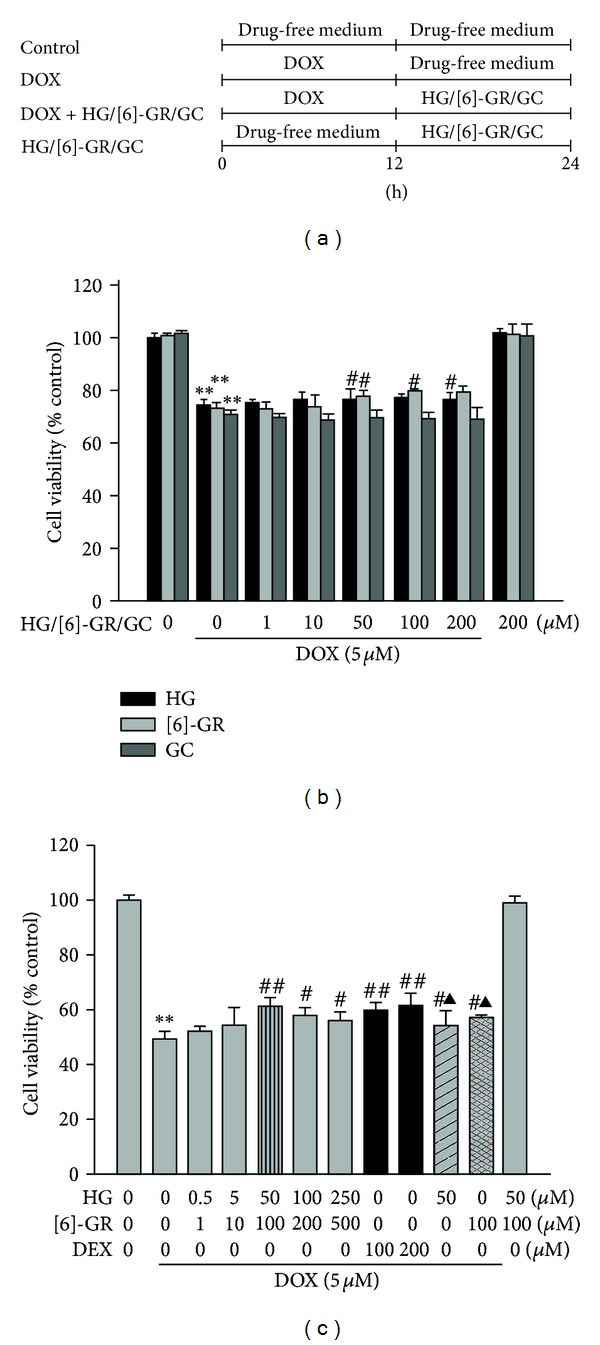
The components of Sini decoction protected NRCs from DOX-induced cell death. (a) Treatment schedules, cells were incubated with or without DOX (5 *μ*M) for 12 h, followed by incubation in drug-free medium or higenamine (HG) or [6]-gingerol ([6]-GR) or glycyrrhizin (GC) for 12 h. (b) Effect of different concentrations of HG or [6]-GR or GC on cell viability in NRCs induced by DOX. (c) Effect of HG/[6]-GR combination, HG single, [6]-GR single, and DEX on cell viability in NRCs induced by DOX. DEX was incubated 1 h prior to DOX at a recommended dose of 10 times or 20 times that of DOX. Cell viability was determined by MTT assay. Results were expressed as percentages of control group. Data are shown as mean ± SD from three independent experiments. **P < 0.01 DOX group versus control group, ^#^ *P* < 0.05, ^##^ *P* < 0.01 versus DOX group. ^▲^ *P* < 0.05 versus HG/[6]-GR combination group.

Cardiomyocytes were isolated from 1–3-day-old Sprague-Dawley rats in accordance with council for International Organizations of Medical Sciences (CIOMS) guidelines and approved by the Animal Care and Use Committee of Sun Yat-sen University (Permit Number: 0111435). NRCs were cultured as previously described with some modifications [21]. Briefly, the minced tissues were serially digested with trypsin (0.05%) and type II collagen (0.07%) in D-Hanks at 37°C. Finally, the harvested cells were incubated in a 10 cm dish at 37°C in a humidified atmosphere (5%CO_2_,95% air) to allow the attachment of noncardiomyocytes. The majority of cardiomyocytes remained in culture medium, which were then collected and cultured in DMEM medium, supplemented with 5% FBS, 15% donor equine serum, and 14% M199. BrdU (0.1 mM) was added to the culture medium for the first 48 h to prevent proliferation of non-cardiomyocytes. After 4 days of culture, cells were incubated in a minimal essential medium (M199 medium supplemented with 1% FBS) overnight before treatment with the indicated procedures. All groups except the control group were exposed to 5 *μ*M DOX for 12 h. Then the nutrient fluid with DOX was removed from the plate and then cultured in the presence or absence of HG, [6]-GR, or GC for another 12 h. The treatment schedule was shown in Figure 2(a).

#### 2.2.2. Cell Viability Assay

Cell viability was monitored by the MTT assay as previously described [22]. Briefly, NRCs were plated at a density of 3 × 10^5^ cells/well in a 96-well plate and routinely incubated for 48 h. After treatment, viable cells were stained with MTT (0.5 mg/mL, 4 h). The supernatant was removed and the formazan crystals were dissolved by the addition of 150 *μ*L of dimethyl sulfoxide (DMSO). Absorbance was measured at 492 nm by an enzyme-linked immunosorbent assay microplate reader (Thermo, Boston, MA, USA). Results were expressed as percentages of control group.

#### 2.2.3. Determination of Oxidative Stress

Dichlorodihydro-fluorescein diacetate (DCFH-DA) is a general oxidative probe that can detect multiple ROS. After indicated treatments, cells were stained with 10 *μ*M DCFH-DA for 30 min at 37°C in the dark. DCFH-DA can be deacetylated in cells, where it can react quantitatively with intracellular radicals to produce the fluorescent dye 20,70-dichlorofluorescein (DCF). Photographs were taken in fluorescence microscope (Leica Microsystems, Bannockburn, IL, USA) after staining with DCFH-DA.

Malondialdehyde (MDA) content, LDH, and superoxide dismutase (SOD) activity were measured by Biochemical Analysis Kit (Jiancheng Biotechnology Co., Nanjing, China) according to protocol instructions, respectively.

#### 2.2.4. Measurement of Mitochondrial Membrane Potential

Mitochondrial membrane potential (MMP) was determined by JC-1. JC-1 exists either as a green fluorescent monomer at depolarized membrane potentials (positive to −100 mV) or as an orange-red fluorescent J-aggregate at hyperpolarized membrane potentials (negative to −140 mV) [23]. The ratio of red-to-green JC-1 fluorescence is dependent only on the MMP. After treatments, cells were incubated with an equal volume of JC-1 (5 *μ*g/mL) at 37°C for 10 min in the dark and rinsed twice with fresh medium without serum. The relative amounts of dual emissions from mitochondrial JC-1 monomers or aggregates were monitored by a laser confocal microscope (ZEISS LSM510 META). The ratios of red/green fluorescent densities from 8 random fields were calculated for each sample. 

#### 2.2.5. Apoptosis Assays

Apoptosis was determined by 4′,6-diamidino-2-phenylindole (DAPI) (Roche, Indianapolis, IN, USA) staining and caspase-3 activation. In the DAPI assay, cells were incubated with 1 *μ*g/mL DAPI-methanol for 15 min at 37°C and analyzed for apoptosis by scoring the percentage of cells having intensely condensed chromatin and/or fragmented nuclei by fluorescence microscopy (Leica Microsystems, Bannockburn, IL, USA). An average of 800–1000 nuclei from 5 random fields was analyzed for each sample. The degree of apoptosis was quantified by an apoptotic index, calculated as the percentage of cells with apoptotic nuclei divided by the number of total cells.

#### 2.2.6. Detection of Cytochrome c Release from Mitochondria

Cytochrome c release was monitored by Western blot analysis after mitochondria/cytosol fractionation. Cells were grown in 10 cm cell culture dishes. After the proper treatment, cells were washed with PBS and incubated with 100 *μ*L of 1.5% digitonin lysis buffer containing 1.5% digitonin, 20 mM Tris-HCl pH 7.4, 140 mM NaCl, 10 mM KCl, and 1 mM MgCl_2_ on ice. After 15 min incubation, the cells were scraped off from the dish and collected in a 600 *μ*L Eppendorf tube. After being placed on ice for approximately 15 min, the extract was then centrifuged for 20 min at 13000 ×g; after that the supernatant was collected as the cytosol fraction. It was stored at −80°C until use. 

#### 2.2.7. Western Blot Analysis

The expression levels of p-Akt (Ser473), total Akt, PI3K, Bcl-2, Bax, cytochrome c, cleaved caspase-3, and procaspase-3 were examined by Western blot analysis. After being treated as previously described, cells were harvested in a lysis buffer containing protein phosphatase inhibitor (Beyotime, China) and protease inhibitor cocktail (Sigma, St. Louis, MO, USA). After centrifugation at 12000 ×g for 15 min at 4°C, the supernatant was analyzed by Western blot. Protein concentration of the extract was determined using Bicinchoninic Acid (BCA) Protein Assay Kit (Kangcheng BioTech, Shanghai, China). An equal amount of protein (60 *μ*g) from each sample was separated by 12% SDS-PAGE and transferred to polyvinylidene fluoride (PVDF) membranes. The membranes were blocked with 5% fat-free dry milk in TBS-T for 1 h at room temperature to prevent nonspecific binding and then incubated with appropriate primary antibodies overnight with gentle agitation at 4°C. Appropriate secondary antibodies conjugated to horseradish peroxidase were then added for 1 h at room temperature. Blots were visualized using ECL (Applygen Technologies Inc., China) or the Li-Cor Odyssey imaging system (Lincoln, NE, USA). The blots were quantified using Image J software (NH, USA).

#### 2.2.8. Quantitative Real-Time PCR for Bcl-2 and Bax mRNA Expression Analyses

Total RNA was extracted from the treated and vehicle control cells with the Trizol reagent according to the manufacturer's instructions. The concentration of total RNA was measured by ultraviolet/visible spectrophotometer (ThermoFisher Scientific, USA). The purity of RNA was estimated by the 260/280 nm absorbance ratio. 1000 ng of total RNA from each sample was used for cDNA synthesis with Prime Script RT reagent Kit (TaKaRa). The primers of Bcl-2, Bax, and *β*-actin were designed as follows: Bax **(NM-017059)** sense: GGTTGCCCTCTTCTACTTT and antisense: AGCCACCCTGGTCTTG, Bcl-2 **(NM-016993) **sense: ACTTTGCAGAGATGTCCG and antisense: CGGTTCAGGTACTCAGCAT, and *β*-actin **(NM-031144)** sense: CGTTGACTCCGTAAAGAC and antisense: TAGGAGCCAGGGCAGTA. The primers used in the qRT-PCR evaluation were specific for every gene as previously reported [24]. cDNA was subsequently amplified with the SYBR Premix Ex TaqTM Kit (TaKaRa) in 8 Strip PCR tubes by the iQ5 instrument (Bio-Rad). Changes in the expression of target genes were measured relative to the mean critical threshold (CT) values of *β*-actin gene [22].

### 2.3. Experiments in H9c2 Cell Line

#### 2.3.1. Cell Culture

Embryonic rat cardiac H9c2 (obtained from American Type Culture Collection) was maintained in DMEM medium supplemented with 10% FBS, 1% penicillin/streptomycin at 37°C under an atmosphere of 5% CO_2_ and 95% air. When cells reached out approximately 70–80% confluence, cells were incubated in a minimal essential medium (DMEM medium supplemented with 1% FBS) overnight before treatment with the indicated procedures.

#### 2.3.2. Cell Viability Assay

Cell viability was detected by CCK-8 (cell counter kit 8). CCK-8 is a sensitive nonradioactive colorimetric assay for determining cell growth (Dojindo Lab., Japan). H9c2 cells were plated onto 96-well plates at a density of 2 × 10^4^ cells/well and incubated at 37°C and humidified 5% CO_2_ until confluence reached 70–80%. After being treated as indicated, CCK-8 solution (10 *μ*L) in 1 : 10 dilution with DMEM (100 *μ*L) was added into each well. Plates were incubated for 2 h at the same incubator conditions after which the absorbance was measured at 450 nm by an enzyme-linked immunosorbent assay microplate reader (Thermo, Boston, MA, USA). Results were expressed as percentages of control group.

#### 2.3.3. FACScan Flow Cytometer Analysis of Cell Apoptosis

Phosphatidylserine (PS) appears on the outer membrane leaflet of cells undergoing programmed cell death. We detected PS exposure on cell plasma membrane using the fluorescent dye Annexin V-FITC Apoptosis Detection Kit (KeyGEN Biotech, China), according to the manufacturer's protocol. This assay can discriminate intact (Annexin V-) and apoptotic (Annexin V+) cells. In brief, cells were harvested and rinsed twice with ice-cold PBS then resuspended in 200 *μ*L binding buffer and incubated with 2 *μ*L of Annexin V-FITC solution for 20 min at room temperature in the dark. Then cells were immediately analyzed by an FACScan flow cytometer (Beckman Coulter, USA).

### 2.4. Statistical Analysis

All data from at least three independent experiments were expressed as the mean ± SD. Statistical comparison among multiple groups was performed by one-way ANOVA followed by least significant difference (LSD) test using the SPSS 13.0 software. *P*  value < 0.05 was considered to be statistically significant.

## 3. Results

### 3.1. Combined Use of HG and [6]-GR Inhibited DOX-Induced Cell Death of NRCs In Vitro

Cell viability was assayed to determine the optimum concentrations necessary for the three components of Sini decoction (HG, [6]-GR, and GC) to protect the NRCs against DOX-induced cytotoxicity. The results demonstrated that HG and [6]-GR increased cell viability in a concentration-dependent manner and the optimal concentrations were 50 *μ*M and 100 *μ*M, respectively. However, GC had no significant protective effect on cell viability (Figure 2(b)). In addition, we found that combined use of HG (50 *μ*M) and [6]-GR (100 *μ*M) exerted more profound protective effects than either drug as a single agent, with effect similar to dexrazoxane (DEX) (100 *μ*M and 200 *μ*M), a clinically approved cardiac protective agent in reducing DOX-induced cardiotoxicity (Figure 2(c)) [25]. In addition, there was no additional protective effect when combined all of the three components (data not shown). Therefore, 50 *μ*M HG and 100 *μ*M [6]-GR were selected for the subsequent in vitro experiments.

### 3.2. Combined Use of HG and [6]-GR Relieved Oxidative Stress Induced by DOX in NRCs

**Figure 3:**
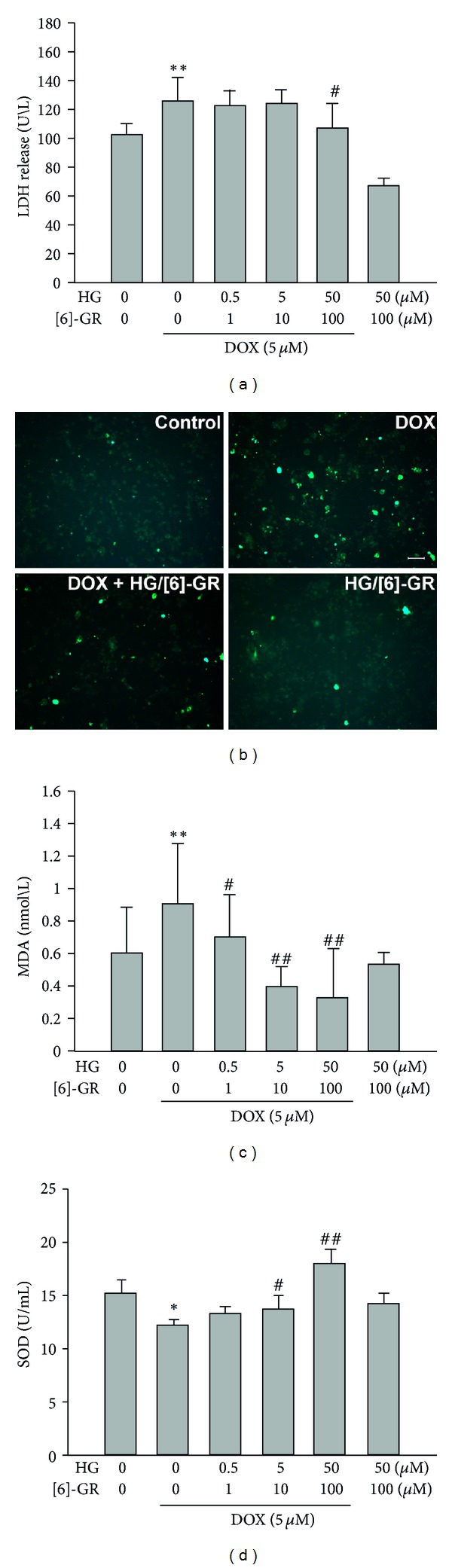
HG/[6]-GR relieved the oxidative stress induced by DOX in NRCs. (a) LDH release in cell supernatant. (b) Staining of intracellular ROS by DCFH-DA in NRCs (original magnification ×100, bar 100 *μ*m). DCFH-DA is a nonfluorescent analog of fluorescein which will emit fluorescence after being oxidized by intracellular ROS. The bright fluorescence was from the highly fluorescent DCF which indicated the concentration and distribution of ROS. (c) MDA content (d) SOD activity. NRCs were preincubated with or without DOX (5 *μ*M) for 12 h and incubated in the presence or absence of HG/[6]-GR for another 12 h. Data were presented as mean ± SD from three independent experiments. *P < 0.05, **P < 0.01 DOX group versus control group, ^#^ *P* < 0.05, ^##^ *P* < 0.01 versus DOX group.

The release of the cytosolic enzyme lactate dehydrogenase (LDH) from cardiomyocytes is commonly used as a measure of doxorubicin and other drug-induced damage [26]. In the present study, DOX exposure significantly increased LDH release in NRCs. Cotreatment with HG and [6]-GR, however, maintained these levels near baseline (Figure 3(a)). 

DOX is a potential source of ROS. The formation of ROS is considered the rate-limiting step in lipid peroxidation. The biochemical determination of malondialdehyde (MDA) indicates lipid peroxide formation [1]. We observed that DOX significantly increased ROS and MDA levels in NRCs compared with control group. However, cotreatment with HG and [6]-GR significantly reduced these levels (Figures 3(b) and 3(c)). The antioxidant enzyme activity (SOD) was illustrated in Figure 3(d). Compared with the control group, DOX-exposed NRCs possessed significantly less SOD activity, whereas treatment with HG plus [6]-GR effectively upregulated SOD activity, even more than that of the control group.

### 3.3. HG/[6]-GR Combination Protected against DOX-Induced Apoptosis in NRCs

**Figure 4:**
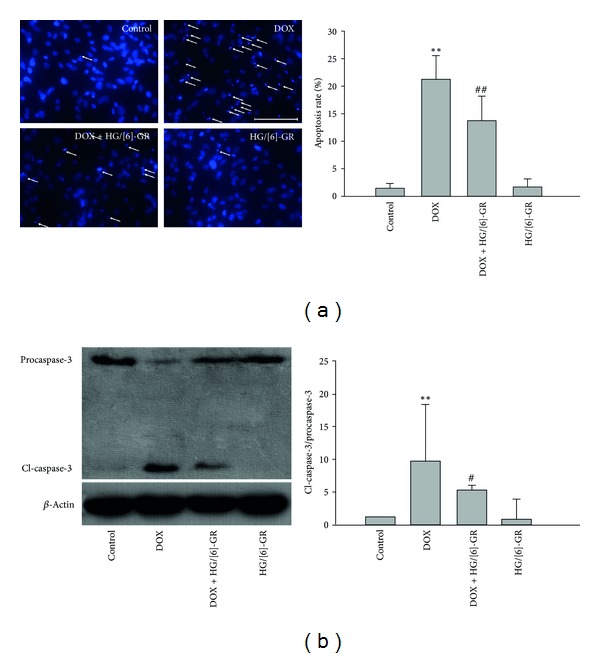
Antiapoptotic effect of HG/[6]-GR in NRCs. NRCs incubated with or without DOX (5 *μ*M) for 12 h, followed by incubation in drug-free medium or HG/[6]-gingerol combination for 12 h. (a) Fluorescence images of DAPI-stained NRCs. The bright parts were the aggregation and fragmentations of chromatin. The condensed and/or irregularly shaped nuclei indicated apoptosis cells (white arrow) (original magnification ×400, bar 100 *μ*m). Quantitative analysis of apoptotic cells by morphological changes and apoptotic nuclear condensation following DAPI staining. (b) Effect of HG/[6]-GR on the expression of caspase-3 activity in NRCs. Cells were similarly treated as indicated. Activation of procaspase-3 was monitored by Western blotting and the relative intensities of protein bands were analyzed by Image J. *β*-Actin was used as an internal control. Experiments were repeated three times and data were shown as mean ± SD. **P < 0.01 DOX group versus control group, ^#^ *P* < 0.05, ^##^ *P* < 0.01 DOX+HG/[6]-GR group versus DOX group.

Apoptosis is a well-known cellular action of DOX. DOX-induced apoptosis via the mitochondrial-mediated intrinsic pathway of apoptosis was assessed by DAPI staining and caspase-3 activation. After DAPI staining, the nuclei in the DOX group appeared either shrunken or irregular. Our data demonstrated that apoptosis cells as identified by DAPI staining were significantly increased in DOX-treated NRCs while the addition of HG/[6]-GR reduced the proportion of these populations (Figure 4(a)). 

As shown in Figure 4(b), the expression of active caspase-3 was significantly increased in DOX group. Treatment with HG/[6]-GR significantly decreased the expression of cleaved caspase-3. However, total recovery from DOX-induced caspase-3 activation was not achieved by HG/[6]-GR combination treatment. 

### 3.4. HG/[6]-GR Combination Suppressed DOX-Induced Disruption of MMP in NRCs

**Figure 5:**
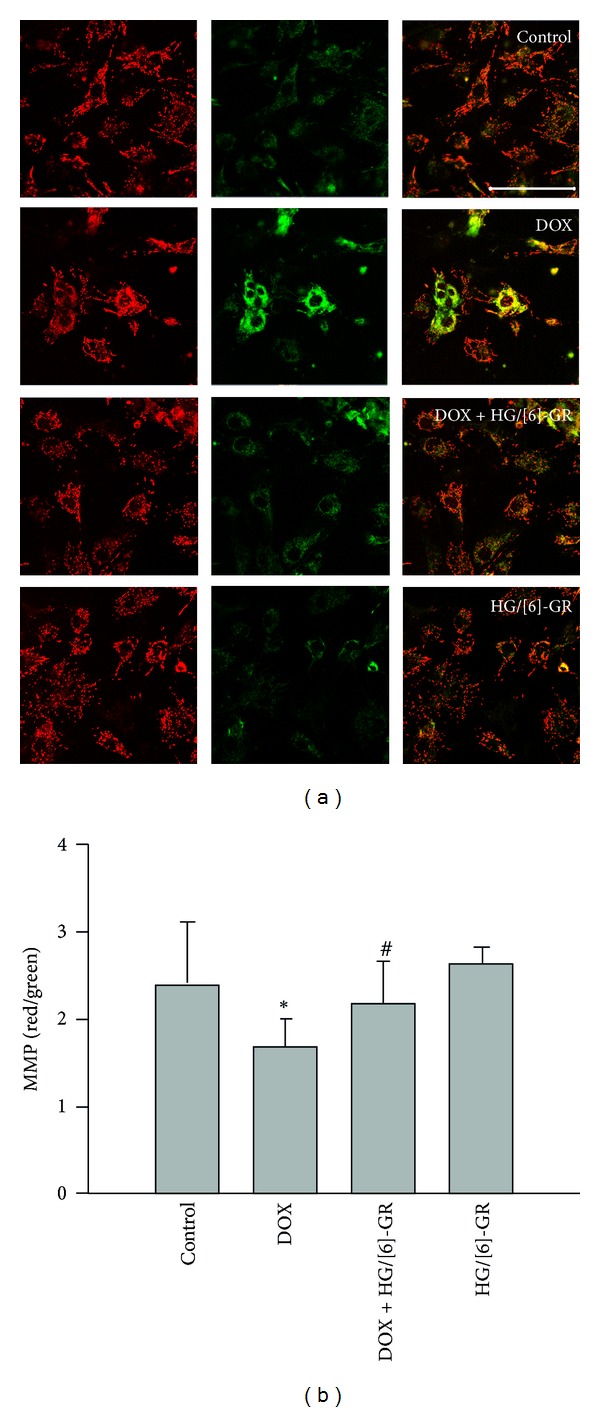
HG/[6]-GR inhibited the collapse of MMP in NRCs induced by DOX. After 24 h of treatment, cells were coincubated with the fluorescence probe JC-1 for 10 min at 37°C; images (original magnification ×400, bar 100 *μ*m) were scanned by confocal laser microscopy and the ratios of red/green fluorescent densities from 8 random fields were calculated for each sample. Data were expressed as mean ± SD. *P < 0.05 DOX group versus control group, ^#^ *P* < 0.05 DOX+HG/[6]-GR group versus DOX group.

Maintenance of intact MMP is critical to cell survival. Stimuli that disrupt mitochondrial potential induce cytochrome c release from mitochondria to the cytosol and trigger a cascade of reactions that lead to cell apoptosis. To determine whether DOX induced apoptosis through disrupting MMP while HG and [6]-GR combination sustained it, we measured MMP by JC-1. Images were scanned by confocal laser microscopy. We observed that MMP was significantly collapsed after exposure to DOX, whereas HG and [6]-GR cotreatment increased MMP (Figure 5), confirming the disruptive effect of DOX and the preservative effect of HG/[6]-GR on MMP.

### 3.5. HG/[6]-GR Combination Increased the Phosphorylation of Akt in NRCs

**Figure 6:**
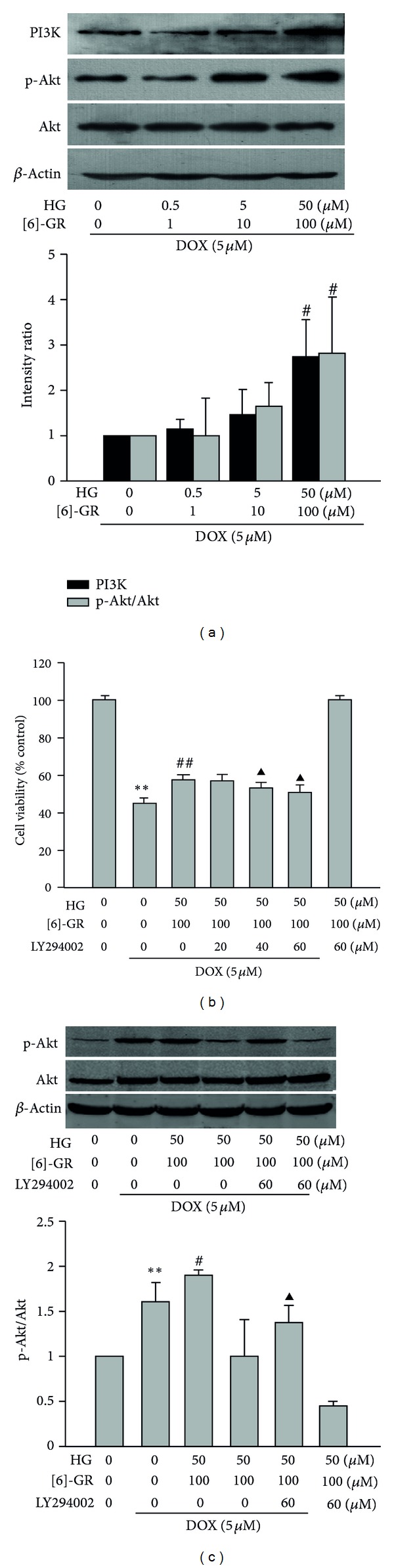
Effect of HG/[6]-GR on p-Akt, Akt, and PI3K expression in NRCs. p-Akt, Akt, and PI3K expressions were detected by Western blot analysis. Cells were preincubated with 5 *μ*M DOX for 12 h and then treated with or without indicated concentrations of HG/[6]-GR for another 12 h. PI3K inhibitor LY294002 was added into the culture medium 60 min before cells lysis or harvest. (a) Representative blots for Akt, p-Akt, and PI3K (upper) and quantitative analysis of the ratio of Akt/p-Akt and PI3K compared to *β*-actin (lower) were given. (b) Effect of HG/[6]-GR on cell viability after addition of LY294002 (60 *μ*M). (c) Effect of HG/[6]-GR on p-Akt after addition of LY294002 (60 *μ*M). Data were shown as mean ± SD from three independent experiments. **P < 0.01 DOX group versus control group, ^#^ *P* < 0.05, ^##^ *P* < 0.01 DOX+HG/[6]-GR group versus DOX group. ^▲^ *P* < 0.05 versus HG/[6]-GR combination group.

A large number of studies have shown that the PI3K/Akt signaling pathway provides an important cell survival signal in cardiomyocytes [1]. Therefore, we used Western blot analysis to detect whether HG/[6]-GR activated PI3K/Akt pathway. As shown in Figure 6(a), HG/[6]-GR upregulated expression of PI3K and p-Akt in DOX-induced NRCs in a dose-dependent manner. Curiously enough, the increased cell activity by HG/[6]-GR was inhibited by LY294002 in DOX-induced NRCs (Figure 6(b)). As expected, the increased level of p-Akt expression due to HG/[6]-GR was significantly suppressed by addition of LY294002 (60 *μ*M), an inhibitor of PI3K (Figure 6(c)).

### 3.6. HG/[6]-GR Combination Upregulated Bcl-2 and Downregulated Bax Expression in NRCs

**Figure 7:**
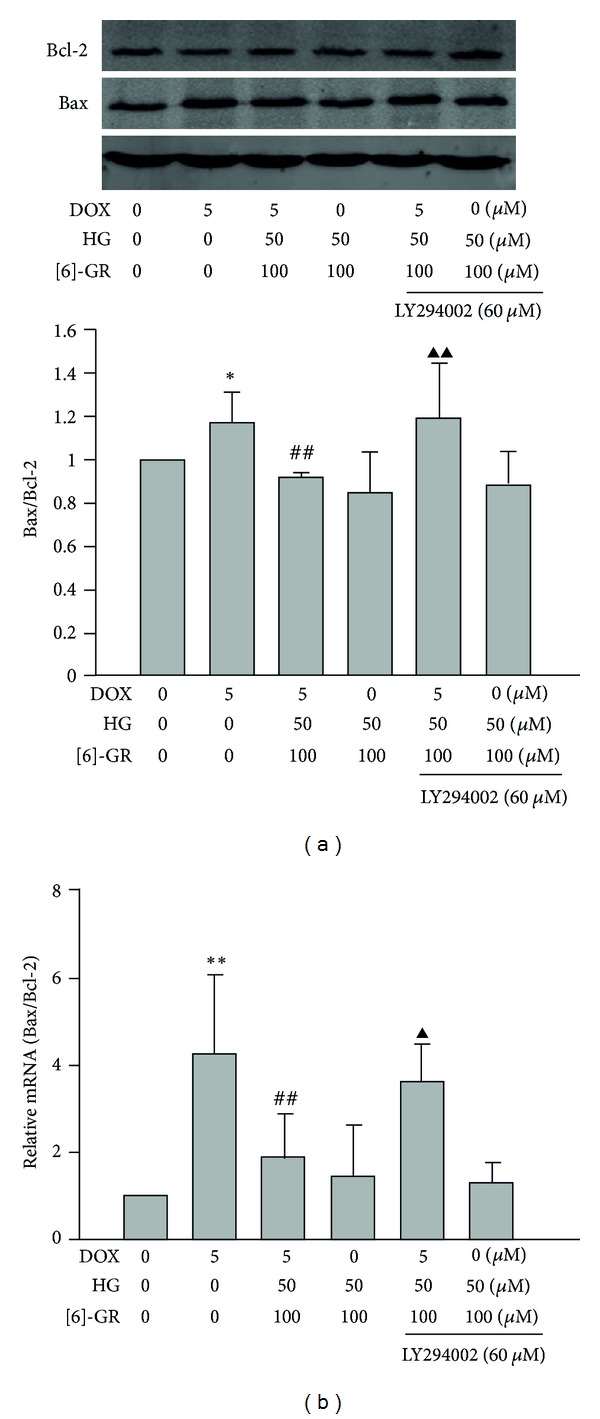
Effect of HG/[6]-GR on the expression of Bcl-2 and Bax in NRCs with the ratio of Bax to Bcl-2 as quantitative analysis. (a) Measurement of protein expression by Western blot. NRCs were preincubated with 5 *μ*M DOX for 12 h and treated with or without HG/[6]-GR for 12 h. Representative blots (upper) and quantitative analysis of Bcl-2 and Bax compared to *β*-actin (lower) were given. (b) Measurement of mRNA by real-time PCR NRCs was similarly treated as indicated. After being treated as indicated, total RNA was subjected to quantitative real-time PCR analysis with specific primers for Bcl-2 and Bax. Experiments were repeated three times and data were shown as mean ± SD (relative to control group) after normalization to *β*-actin. *P < 0.05, **P < 0.01 DOX group versus control group, ^##^ *P* < 0.01 DOX+HG/[6]-GR group versus DOX group. ^▲^ *P* < 0.05, ^▲▲^ *P* < 0.01  versus HG/[6]-GR combination group.

Western blot analysis of the expression of proapoptotic or antiapoptotic proteins showed that DOX significantly increased the expression of proapoptotic Bax protein but decreased Bcl-2 protein in NRCs. However, by the treatment of HG/[6]-GR, the expression of Bax was downregulated while Bcl-2 was upregulated; thus the ratio of Bax/Bcl-2 was decreased (Figure 7(a)). It should be noted that the decreased ratio of Bax/Bcl-2 by HG/[6]-GR was significantly counteracted by LY294002.

Quantitative real-time PCR analysis data further supported that DOX increased pro-apoptotic gene expression (Bax) but decreased anti-apoptotic genes (Bcl-2). Compared with control group, the ratio of Bax/Bcl-2 increased by 327% (*P* < 0.001); however, these effects were reversed by the treatment of HG/[6]-GR. As expected, addition of LY294002 significantly inhibited the effect of HG/[6]-GR on the ratio of Bax/Bcl-2 (Figure 7(b)).

### 3.7. HG/[6]-GR Combination Inhibited the Mitochondria-Mediated Cardiomyocytes Apoptosis Induced through PI3K/Akt by DOX in NRCs

**Figure 8:**
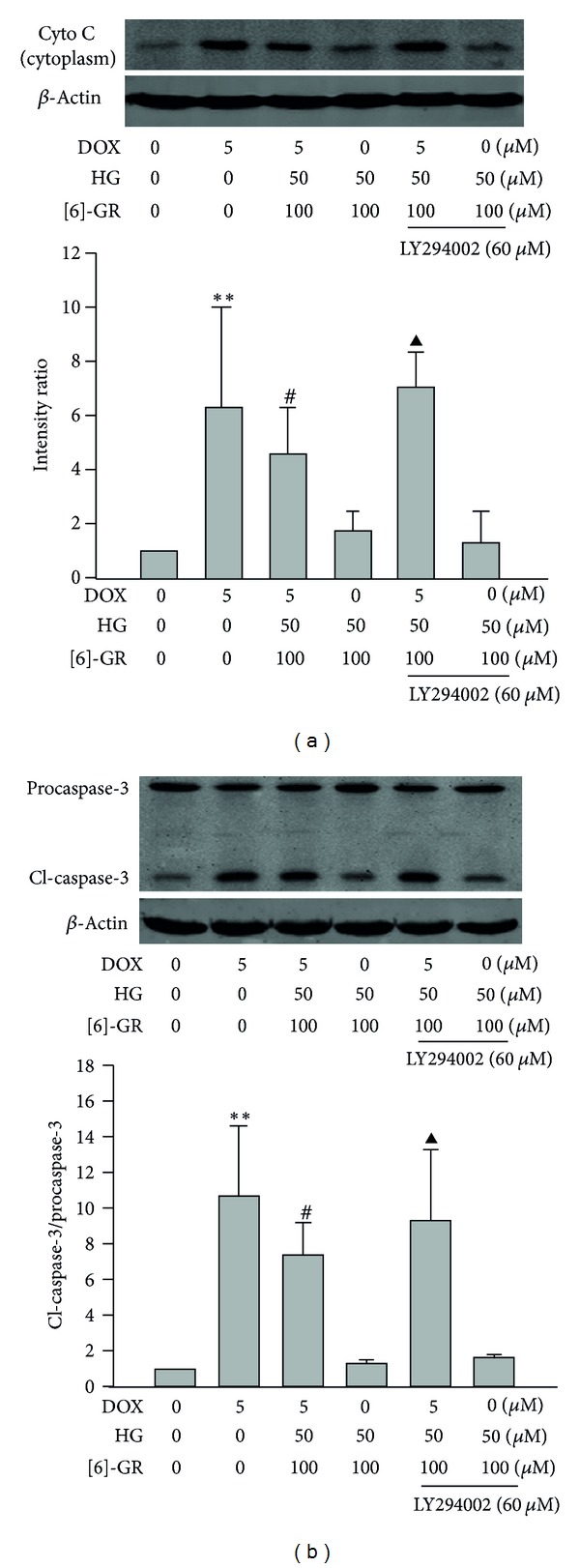
HG/[6]-GR inhibited DOX-induced apoptosis via activation of Akt in NRCs. Effect of HG/[6]-GR on cell viability after addition of PI3-kinase inhibitor (LY294002). Effect of HG/[6]-GR on cytochrome c (a) and cleaved caspase-3 expression (b) after addition of LY294002. PI3K inhibitor LY294002 was added into the culture medium 60 min before cells lysis. Cell lysate was blotted with cytochrome c, cl-caspase-3, and procaspase-3. Scanning densitometry was used for semiquantitative analysis compared to *β*-actin level. Experiments were repeated three times and data were shown as mean ± SD. **P < 0.01 DOX group versus control group, ^#^ *P* < 0.05 DOX+HG/[6]-GR group versus DOX group. ^▲^ *P* < 0.05 DOX+HG/[6]-GR+LY294002 group versus DOX+ HG/[6]-GR combination group.

As shown in Figure 8(a), most of detectable cytochrome c was found in the cytosol fraction in DOX group. Accordingly, addition of HG/[6]-GR resulted in a decrease of the expression of cytochrome c in cytosol fraction, which was significantly reversed by treatment with LY294002. Cardiomyocytes apoptosis was also studied in terms of active caspase-3, a key downstream effectors protein of apoptosis. It should be noted that the decreased expression of active caspase-3 by HG/[6]-GR was significantly counteracted by LY294002 (Figure 8(b)).

### 3.8. HG/[6]-GR Combination Protected against DOX-Induced Cell Death of H9c2 Cells: MTT Assay, Damage of MMP, and Annexin V Staining in DOX-Treated H9c2 Cells

**Figure 9:**
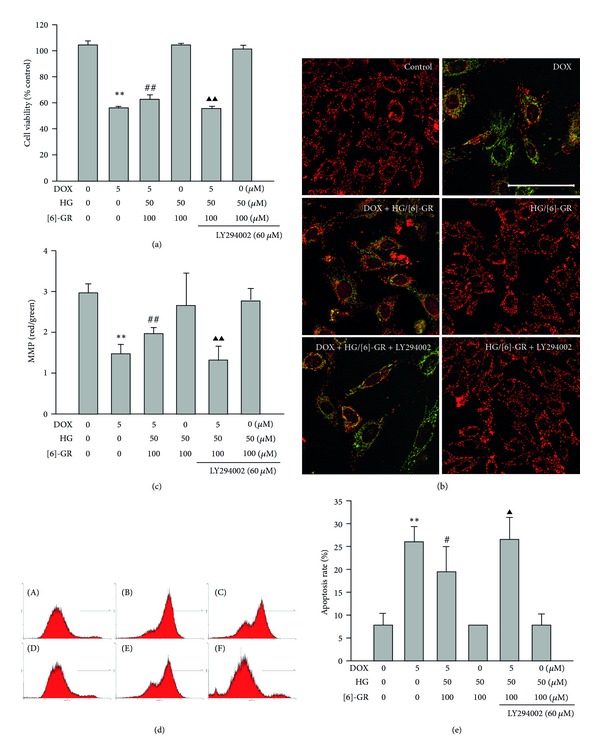
HG/[6]-GR inhibited DOX-induced apoptosis or cell death via activation of Akt in H9c2 cells. (a) Effect of HG/[6]-GR on cell viability after addition of PI3-kinase inhibitor (LY294002). ((b) and (c)) effect of HG/[6]-GR on MMP after addition of LY294002 (original magnification ×400, bar 100 *μ*m). ((d) and (e)) flow histogram analysis of Annexin V staining. (A) control group, (B) DOX group, (C) DOX+HG/[6]-GR group, (D) HG/[6]-GR group; (E) HG/[6]-GR+LY294002 group, (F) DOX+HG/[6]-GR+LY294002 group. Cells were pretreated with 5 *μ*M DOX for 12 h and then incubated with or without indicated concentrations of HG/[6]-GR for another 12 h. PI3K inhibitor LY294002 was added into the culture medium 60 min before cells harvest. Experiments were repeated at least three times and data were shown as mean ± SD. *P < 0.05, **P < 0.01 DOX group versus control group, ^#^ *P* < 0.05, ^##^ *P* < 0.01 DOX+HG/[6]-GR group versus DOX group. ^▲^ *P* < 0.05, ^▲▲^ *P* < 0.01 DOX+HG/[6]-GR+LY294002 group versus DOX+ HG/[6]-GR combination group.

In support of the protective role of HG/[6]-GR, we examined its role in H9c2 cells which were exposed to 5 *μ*M DOX for 12 h. Similarly, we found that DOX exposure decreased the number of viable cells (Figure 9(a)), disrupted MMP (Figures 9(b) and 9(c)), and induced apoptotic cell death (Figures 9(d) and 9(e)). These alterations in cell viability, MMP, and apoptosis due to DOX exposure were attenuated by treatment with HG/[6]-GR, further affirming the proapoptotic action of DOX and the antiapoptotic role of HG/[6]-GR. Similarly, the cytoprotection of HG/[6]-GR was abolished by LY294002, suggesting that once again HG/[6]-GR exerted cardioprotective effect against DOX injury via activation of the PI3K/Akt signaling pathway.

## 4. Discussion

Our data clearly showed that DOX induced intracellular oxidative stress, activated mitochondria-dependent apoptotic pathway, and stimulated cardiomyocytes apoptosis in vitro models of H9c2 cell line and NRCs. However, HG/[6]-GR cotreatment markedly attenuated DOX-induced oxidative stress and cardiomyocytes apoptosis. The possible mechanism explaining the beneficial effects of HG/[6]-GR combination may involve the activating of PI3K/Akt signaling pathway. These results suggested that HG and [6]-GR may be the active components of Sini decoction and it can be used as a cytoprotective agent in DOX chemotherapy. 

A number of studies have been undertaken to find adjuvant therapies with the ability to prevent DOX-induced cardiomyopathy. Previous in vivo and clinical studies performed by our group found that Sini decoction possessed protective effects against cardiovascular disease [27–29]. In recent years, we found that Sini decoction could protect against DOX-induced heart failure, and the mechanism may be involved in antiapoptotic effect and antioxidative activity [9, 10]. However, the active components of Sini decoction are still unclear due to its complex components. It was reported that HG reduced rat I/R-induced myocardial damage through HO-1-dependent mechanism [14]. Recently, it was shown that [6]-GR protected against DOX-induced cardiotoxicity through its antioxidative effect and modulation of NF-*κ*B as well as apoptosis [30]. Moreover, research showed that glycyrrhizinate could ameliorate rabbit myocardial ischemia-reperfusion injury through P38MAPK pathway [31]. Thus, it is of great interest to investigate therapeutic potential of these chemicals in DOX-induced disorders.

 Dexrazoxane (DEX) is the only well-established and clinically approved agent used in cancer patients to prevent DOX-mediated cardiotoxicity. DEX provides cardiac protection from anthracycline primarily through its hydrolytic products, which have the ability to remove iron from iron/DOX complexes and thus to reduce the formation of reactive oxygen radicals [32]. In this study, The effects of HG/[6]-GR combination on cell viability were comparable to those observed with DEX, added at a recommended dose of 10 times or 20 times that of DOX [33]. In addition, HG combined with [6]-GR was highly effective in protecting cardiomyocytes from DOX-induced LDH release, which further confirmed that HG/[6]-GR combination suppressed cell death of cultured cardiomyocytes induced by DOX in vitro.

DOX-induced cardiotoxicity is a complex multifactorial process, in which mitochondrial ROS production plays a critical role [34]. DOX is a potential source of ROS, the formation of which is considered the rate-limiting step in lipid peroxidation. The biochemical determination of MDA indicates the formation of lipid peroxide [1]. Antioxidant enzyme activities (SOD) reflect the level of oxidative stress. Weak antioxidant capacity in the heart may be a factor responsible for the high sensitivity of this organ to DOX-induced oxidative damage [35]. Although the relationship among the events of DOX-induced cytotoxicity, ROS generation, and apoptosis is not well defined, oxidative stress can induce cardiomyocytes apoptosis in vitro. The ability of HG/[6]-GR to scavenge free radicals, to reduce MDA formation, and to upregulate SOD activity may contribute to the protective role of reducing cardiomyocytes apoptosis from DOX injury.

Cardiomyocytes apoptosis is one of the most important pathogenic mechanisms underlying DOX damage. Any loss through cell apoptosis due to anticancer drugs will create a deficit of contractile elements, thus leading to cardiac dysfunction [36]. Inhibition of cardiomyocytes apoptosis could prevent the loss of contractile cells and minimize cardiac damage induced by DOX. Mitochondria are not only critical for the generation of energy but also critical for apoptosis. The preservation of mitochondrial integrity contributed to the prevention of apoptosis [37]. It is clear that mitochondria are a likely target of DOX; DOX accumulates in mitochondria are able to depolarize the MMP [38]. The collapse of MMP results in the release of cytochrome c from the intermembrane space into the cytosol [39]. Once released cytochrome c binds to the apoptotic protease-activating factor-1 (Apaf-1) and assembles into a heptamer structure in the presence of ATP, it promotes the activation of pro-caspase-9 [40]. Once activated, caspase-9 then presumably triggers a cascade of caspase activation events to execute the cell death program. We found that DOX depolarized MMP, led to translocation of cytochrome c from mitochondria to cytosol, and activated caspase-3 to execute the cell death program while HG/[6]-GR was able to retain MMP and inhibit this process. 

Many genes have been reported to be linked with the regulation of programmed cell death under physiological and pathological conditions, in which Bcl-2 and Bax genes are suggested to play a major role in determining cell's survival or death after apoptotic stimuli [14]. Bcl-2 regulates mitochondria-dependent pathway by interfering with the release of cytochrome c or binding to Apaf-1 through its interaction with Bax [41, 42]. When Bax predominates, programmed cell death is accelerated, and the death repressor activity of Bcl-2 is countered; therefore, Bax/Bcl-2 ratio is often adopted to represent the extent of apoptosis [43, 44]. As expected, HG/[6]-GR significantly reduced the ratio of Bax/Bcl-2 at the mRNA and protein levels in those cardiomyocytes that were subjected to DOX. We believe that the antiapoptotic effect of HG/[6]-GR is due to modulation of Bcl-2/Bax by HG/[6]-GR. How is it possible for HG/[6]-GR to modulate these genes? 

We speculate that PI3K/Akt signaling pathway is responsible for this, because upegulation of p-Akt expression markedly reduced the apoptotic cell death due to DOX-induced injury in vitro and the effect was completely abolished by PI3K inhibitor [25]. Indeed, a great number of studies have shown that PI3K/Akt signaling pathway provided an important cell survival signal in cardiomyocytes. Activation of the PI3K/Akt pathway attenuated mitochondria-mediated apoptosis [42, 43, 45, 46]. DOX-induced damage and apoptosis of cardiomyocytes have been associated with downregulation of Akt in vitro and in vivo animal models [13]. In this study, we found that the expression of p-Akt was upregulated by DOX, which might be a compensatory self-protective effect. After being treated with HG/[6]-GR, the expression of p-Akt was increased compared with DOX group. We found that the upregulation of p-Akt protein occurring in cardiomyocytes after being treated with HG/[6]-GR was related to the reduction of apoptosis index, Bax gene expression, cytochrome c release, and caspase-3 activity. Moreover, the beneficial effects exerted by HG/[6]-GR in DOX-induced cytotoxicity models such as reduction of cytochrome c release and caspase-3 activity were suppressed by the presence of LY294002, which clearly suggest that Akt was required for the protective mechanism of HG/[6]-GR. 

Combination therapy has been advocated for more than 2,500 years by prescriptions called formulae in traditional Chinese medicine, a unique medical system assisting the ancient Chinese in dealing with disease. It is believed that, at least in some formulae, multiple components could hit multiple targets and exert synergistic therapeutic efficacies [47]. Sini decoction with long history of use has been proven to be effective in treating cardiovascular disease. Analyzing the active components of Sini decoction after treatment by DOX in vitro or in vivo may help dissect its underlying efficacies and mechanisms of action and exploit new ideal drugs ultimately.

In conclusion, we have presented data which, to our knowledge, provides the first demonstration that HG and [6]-GR may be the major active component of Sini decoction, and HG/[6]-GR combination can promote the survival of H9c2 cell line and NRCs subjected to DOX. This potentially beneficial effect may involve antioxidative effects and attenuation of the intrinsic apoptotic pathway, possibly via the PI3K/Akt signaling pathways.

## Abbreviations

DOX: Doxorubicine
HG: Higenamine
[6]-GR: [6]-Gingerol
GC: Glycyrrhizin
NRCs: Neonatal rat cardiomyocytes
FBS: Fetal bovine serum
M199: Medium 199
DMEM: Dulbeccos modified eagle medium
PBS: Phosphate-buffered saline
MMP: Mitochondrial membrane potential
PI3K: Phosphoinositide 3-kinase
P-Akt: Phospho-Akt
Akt: Protein kinase B
DEX: Dexrazoxane;
LDH: Lactate dehydrogenase
MDA: Malondialdehyde
SOD: Superoxide dismutase
HG/[6]-GR: HG plus [6]-GR
ROS: Reactive oxygen species
PS: Phosphatidylserine.
